# A Review of Analytical and Chemometric Strategies for Forensic Classification of Homemade Explosives

**DOI:** 10.1002/ansa.70010

**Published:** 2025-04-16

**Authors:** Abdulrahman Aljanaahi, Muhammad K. Hakeem, Abdulla Aljanaahi, Iltaf Shah

**Affiliations:** ^1^ Dubai Police General Headquarters Dubai UAE; ^2^ Department of Chemistry College of Science United Arab Emirates University (UAEU) Al Ain UAE

**Keywords:** chemometric methods, explosive residue detection, forensic analysis, gas chromatography–mass spectrometry (GC–MS), homemade explosives (HMEs), infrared spectroscopy (IR)

## Abstract

Homemade explosives (HMEs), commonly used in improvised explosive devices (IEDs), present a significant forensic challenge due to their chemical variability, accessibility and adaptability. Traditional forensic methodologies often struggle with environmental contamination, complex sample matrices and the non‐specificity of precursor residues. Recent advances in analytical techniques and chemometric methods have enhanced the detection, classification and interpretation of explosive residues. Infrared (IR) spectroscopy and gas chromatography–mass spectrometry (GC–MS) have seen improvements in spectral resolution and real‐time detection capabilities, allowing for more accurate differentiation of explosive precursors. Thermal analysis techniques, such as thermogravimetric analysis (TGA) and differential scanning calorimetry (DSC), now provide refined kinetic modelling to assess the decomposition pathways of unstable energetic materials, improving forensic risk assessments. Additionally, x‐ray diffraction (XRD) has contributed to forensic material sourcing by distinguishing between industrial‐grade and improvised explosive formulations. Chemometric approaches, including principal component analysis (PCA), linear discriminant analysis (LDA) and partial least squares discriminant analysis (PLS‐DA), have revolutionized forensic data analysis by improving classification accuracy and enabling automated identification of explosive components. Advanced machine learning models are being integrated with spectral datasets to enhance real‐time decision‐making in forensic laboratories and portable field devices. Despite these advancements, challenges remain in adapting laboratory‐based techniques for field deployment, particularly in enhancing the sensitivity and robustness of portable analytical instruments. This review critically evaluates the latest developments in forensic analytical chemistry, highlighting strengths, limitations and emerging strategies to improve real‐world HME detection and classification.

## 1 Introduction

The proliferation of improvised explosive devices (IEDs) has emerged as a significant security threat, posing substantial risks to public safety, infrastructure and national security. The accessibility of explosive precursor materials, coupled with the ease of assembly, makes IEDs a versatile and widespread threat that transcends geopolitical and economic boundaries. Their use is not limited to conflict zones but extends to urban and civilian environments, complicating counterterrorism and forensic efforts. The consequences of IEDs are far‐reaching, impacting not only immediate casualties but also economic stability, environmental safety and international diplomatic relations [1, 2, 3]. Homemade explosives (HMEs), a critical subset of IEDs, present unique challenges due to their diverse chemical compositions and ease of synthesis. Recent studies categorize HMEs based on their precursor materials, including peroxide‐based explosives (e.g., triacetone triperoxide, TATP), nitrate‐based explosives (e.g., ammonium nitrate [AN] fuel oil, ANFO) and chlorate‐based explosives (e.g., potassium chlorate mixtures). Each category exhibits distinct thermal stability, decomposition behaviour and structural properties, complicating forensic investigations [4, 5, 6, 7].

Additionally, the widespread availability of precursor chemicals in household, agricultural and industrial products makes the tracking and regulation of these materials highly challenging. Environmental contamination, variability in synthesis methods and the presence of impurities further complicate forensic analysis, making accurate detection and classification difficult. The forensic analysis of HMEs is vital for identifying their chemical signatures, linking residues to their sources and developing preventive measures against their misuse. The increasing frequency of terrorist activities and criminal use of HMEs underscores the urgency of refining analytical methodologies for their detection and characterization. Consequently, advancements in analytical chemistry have become paramount in ensuring accurate forensic identification, origin‐tracing and classification of explosive materials [8].

## 2 Advances in Analytical Technologies

The forensic detection and analysis of HMEs require advanced analytical techniques capable of distinguishing explosive compounds from benign materials with high sensitivity and specificity. Infrared (IR) spectroscopy and gas chromatography–mass spectrometry (GC–MS) have been widely used for identifying the chemical signatures of HMEs [9]. These techniques provide molecular‐level insights into explosive compositions, enabling forensic experts to differentiate among various explosive formulations. However, despite their effectiveness, current forensic methodologies face challenges related to environmental contamination, matrix effects and the reproducibility of results in field conditions.

Innovative tools, such as laser‐driven thermal reactors, near‐infrared (NIR) spectroscopy and high‐resolution mass spectrometry (HRMS), are being explored to enhance the forensic detection of explosives. However, traditional analytical methods still struggle to differentiate HMEs from chemically similar non‐explosive substances, leading to false positive or inconclusive results. Environmental contamination further complicates forensic investigations by altering chemical signatures and introducing variability in spectral data [10, 11, 12, 13, 14].

Another major challenge is the limited portability of sophisticated forensic instruments. Although laboratory‐based methods provide high accuracy, the need for real‐time, field‐deployable forensic tools is growing. The integration of chemometric approaches with portable analytical systems has the potential to enhance data interpretation and classification accuracy in dynamic environments. Additionally, ensuring the robustness and reproducibility of forensic methods across different operational conditions remains a key concern. Recent reviews, such as López‐López and García‐Ruiz [15], have discussed analytical techniques for general explosives detection, focusing on trace detection and safety protocols [15]. However, this review uniquely emphasizes the fusion of analytical techniques with chemometric methodologies tailored for HME classification. By addressing critical challenges, such as method reproducibility, field applicability and data processing limitations, this review aims to provide a structured roadmap for advancing forensic methodologies. The discussion extends beyond conventional detection methods to highlight the integration of artificial intelligence (AI), machine learning (ML) and advanced chemometric techniques for improving forensic capabilities.

Given the persistent and evolving threats posed by HMEs, continuous research is essential for refining detection technologies, improving field applicability and enhancing forensic reliability. This review critically evaluates recent advancements in analytical and chemometric techniques for forensic HME analysis, identifies existing limitations and proposes future research directions aimed at strengthening forensic capabilities in real‐world applications.

### 2.1 IR in Forensic Analysis of Explosive Materials

IR spectroscopy is a crucial analytical technique in forensic science, widely used for detecting and characterizing explosive materials. By analysing molecular vibrations, IR spectroscopy offers a non‐destructive and highly specific method for forensic investigations. Advanced IR methodologies such as Fourier‐transform infrared (FTIR) spectroscopy, attenuated total reflectance FTIR (ATR‐FTIR) spectroscopy, optical‐photothermal infrared (O‐PTIR) spectromicroscopy and NIR spectroscopy have enhanced forensic capabilities in detecting and classifying explosives with improved sensitivity and specificity [16–15, 18, 19, 20]. The samples analysed in many studies underwent preparation steps to improve spectral accuracy. These steps included drying, homogenizing and filtering to remove contaminants and ensure consistency across measurements.

**FIGURE 1:**
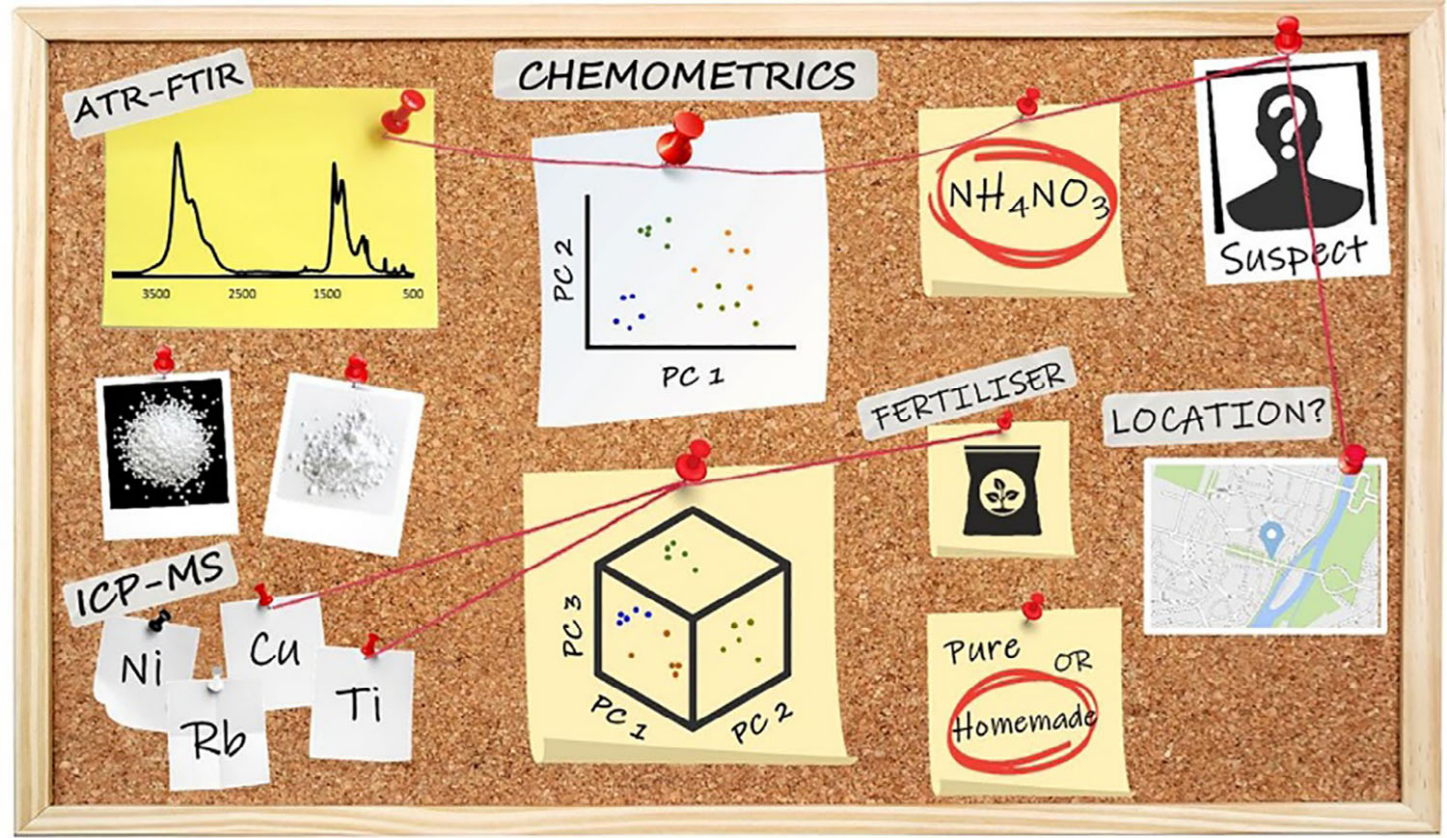
Graphical abstract illustrating the forensic source determination of ammonium nitrate (AN). *Source*: Figure adapted from D'Uva et al. [16], Copyright 2022, Elsevier.

Although FTIR spectroscopy provides high‐resolution molecular fingerprints, ATR‐FTIR is increasingly favoured for its superior surface sensitivity and minimal sample preparation requirements. In a study by D'Uva et al. [16], ATR‐FTIR, in conjunction with trace elemental analysis via inductively coupled plasma mass spectrometry (ICP‐MS) and chemometric modelling, was employed for a comprehensive forensic analysis of AN products. The study examined both pure AN and homemade AN formulations, achieving a 92.5% classification accuracy using a discriminant function model. Further refinement through stepwise linear discriminant analysis (LDA) and principal component analysis (PCA) enabled clear differentiation between pure and homemade AN samples, with ATR‐FTIR sulphate peaks and trace elemental variations emerging as key discriminators. Although some overlap in sample clusters was noted, the integration of ATR‐FTIR, ICP‐MS and chemometric tools provided a robust and validated approach for forensic source determination of AN, essential in explosives investigations [16]. A schematic representation of this forensic workflow is depicted in Figure 1, highlighting the analytical synergy among ATR‐FTIR, ICP‐MS and multivariate data analysis.

Another important development is the use of multivariate analysis techniques in post‐blast residue examination, where residue particles were dissolved and filtered to enhance spectral clarity. Banas et al. [17] demonstrated the effectiveness of hierarchical cluster analysis (HCA) and PCA in enhancing the spectroscopic classification of explosive residues. By employing chemometric approaches, forensic experts can distinguish explosive components from environmental contaminants with improved precision [17]. Ewing and Kazarian [18] elaborated on the value of IR spectroscopy and spectroscopic imaging in forensic science. Their review underscored the potential of FTIR spectroscopic imaging in various modes, including transmission, external reflection and ATR, for forensic applications. These techniques allow detailed chemical profiling of forensic samples. Such advancements have enabled forensic investigators to detect explosive residues even within fingerprint evidence, aiding in suspect identification [18]. López‐López and García‐Ruiz [15] provided a comprehensive review of recent advances in IR and Raman spectroscopy for explosives analysis. Their study highlighted the critical role of these techniques in homeland security and environmental monitoring, particularly in the trace detection and characterization of explosive substances [15].

**FIGURE 2:**
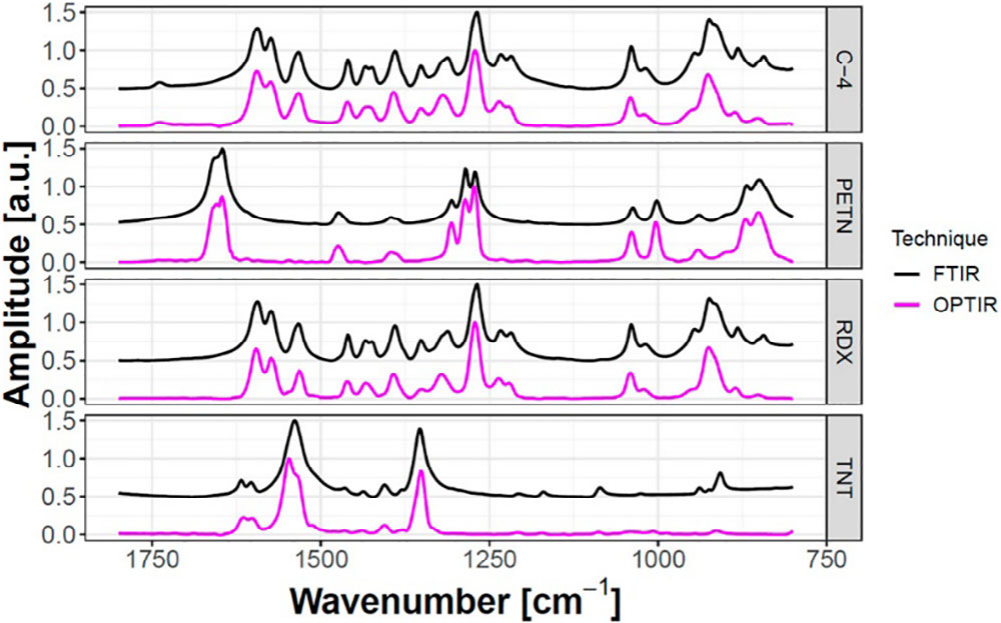
Comparison of FTIR (black) and O‐PTIR (pink) spectra for high explosives (C‐4, RDX, PETN and TNT), showing spectral matching. FTIR, Fourier‐transform infrared spectroscopy; O‐PTIR, optical‐photothermal infrared; PETN, pentaerythritol tetranitrate; RDX, Royal Demolition Explosive; TNT, 2,4,6‐trinitrotoluene. *Source*: Figure adapted from Banas et al. [19], Copyright 2020, ACS Analytical Chemistry.

Furthermore, O‐PTIR spectromicroscopy, as demonstrated by Banas et al. [19], provides a non‐destructive approach for detecting high‐explosive materials within fingerprints. Figure 2 compares O‐PTIR and FTIR spectra, highlighting O‐PTIR's superior forensic potential in identifying high explosives.

**FIGURE 3:**
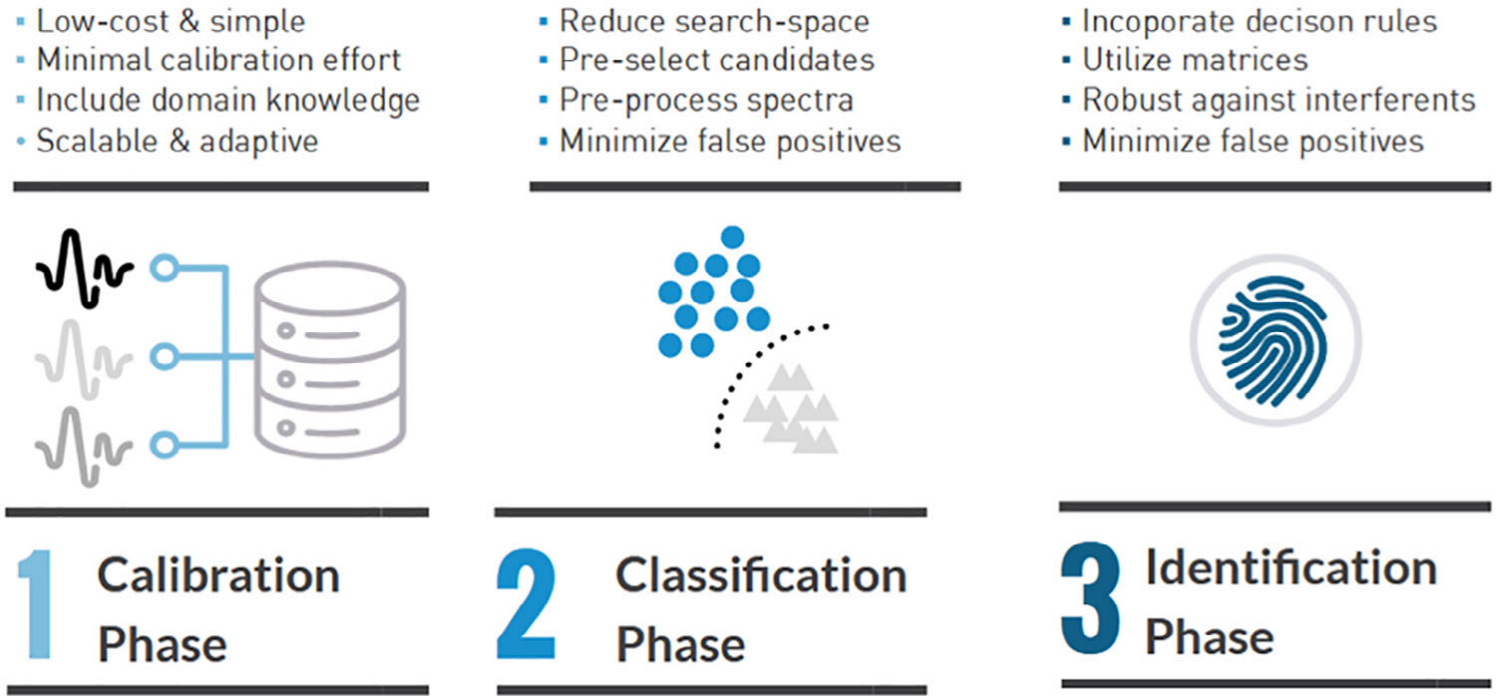
Chemometric model for NIR‐based forensic identification of intact energetic materials, integrating an explosives reference matrix, LDA classification and NAS‐based spectral reconstruction. *Source*: Figure adapted from Van Damme et al. [20], published under the CC BY license (MDPI, Sensors).

This novel method overcomes the limitations of traditional IR techniques by offering higher spatial resolution and eliminating fluorescence interference, making it an ideal candidate for forensic investigations [19]. One of the major limitations of laboratory‐based IR techniques is their inability to be deployed in the field. Recent advancements in portable NIR spectroscopy have bridged this gap. Van Damme et al. [20] demonstrated the feasibility of NIR spectroscopy combined with multivariate data analysis for on‐site identification of intact energetic materials, providing real‐time forensic insights, as shown in Figure 3. This approach enables real‐time and non‐invasive detection of a broad spectrum of explosive materials, significantly enhancing on‐site forensic capabilities [20].

Despite these advancements, forensic IR spectroscopy faces challenges in analysing complex mixtures, quantifying trace explosives and addressing spectral overlaps caused by contaminants in post‐blast residues. Additionally, portable IR technologies still require improvements in sensitivity and robustness to match laboratory‐based techniques. A comparative analysis of these IR spectroscopy techniques, including their advantages and limitations in forensic explosive analysis, is summarized in Table 1. Future research should focus on enhancing chemometric integration in IR‐based forensic analysis for better spectral interpretation, developing miniaturized, high‐sensitivity portable IR devices for on‐site explosives detection and improving quantitative IR methods for forensic validation and legal admissibility.

**TABLE 1:** Comparative analysis of infrared spectroscopy techniques in forensic explosive analysis.

IR technique	Advantages	Limitations
FTIR	High‐resolution molecular fingerprinting; well‐established forensic method	Requires sample preparation; interference from environmental contaminants
ATR‐FTIR	Minimal sample preparation; high surface sensitivity; effective for solid‐phase analysis	Limited penetration depth; sensitivity varies based on sample homogeneity
O‐PTIR	High spatial resolution; overcomes fluorescence issues; suitable for fingerprint analysis	Requires advanced instrumentation; not widely available in forensic labs
NIR spectroscopy	Portable, rapid on‐site detection; effective for field applications	Lower spectral resolution compared to FTIR; requires chemometric models for data interpretation

Abbreviations: ATR‐FTIR, attenuated total reflectance Fourier‐transform infrared; FTIR, Fourier‐transform infrared; IR, infrared; NIR, near‐infrared; O‐PTIR, optical‐photothermal infrared.

### 2.2 Thermogravimetric Analysis (TGA) in Forensic Analysis of Explosives

TGA is a fundamental thermal technique in forensic investigations, widely utilized for assessing the thermal stability, decomposition behaviour and composition of explosive materials. By continuously measuring the weight loss of a substance under controlled temperature increases, TGA provides crucial data for understanding the thermal degradation pathways of explosives. This capability is essential in forensic science for classifying explosives based on decomposition kinetics, identifying unknown explosive materials and ensuring safe handling and storage protocols. The technique has been particularly valuable in differentiating commercial and HMEs by examining their thermal signatures under controlled atmospheres [21, 22, 23].

**FIGURE 4:**
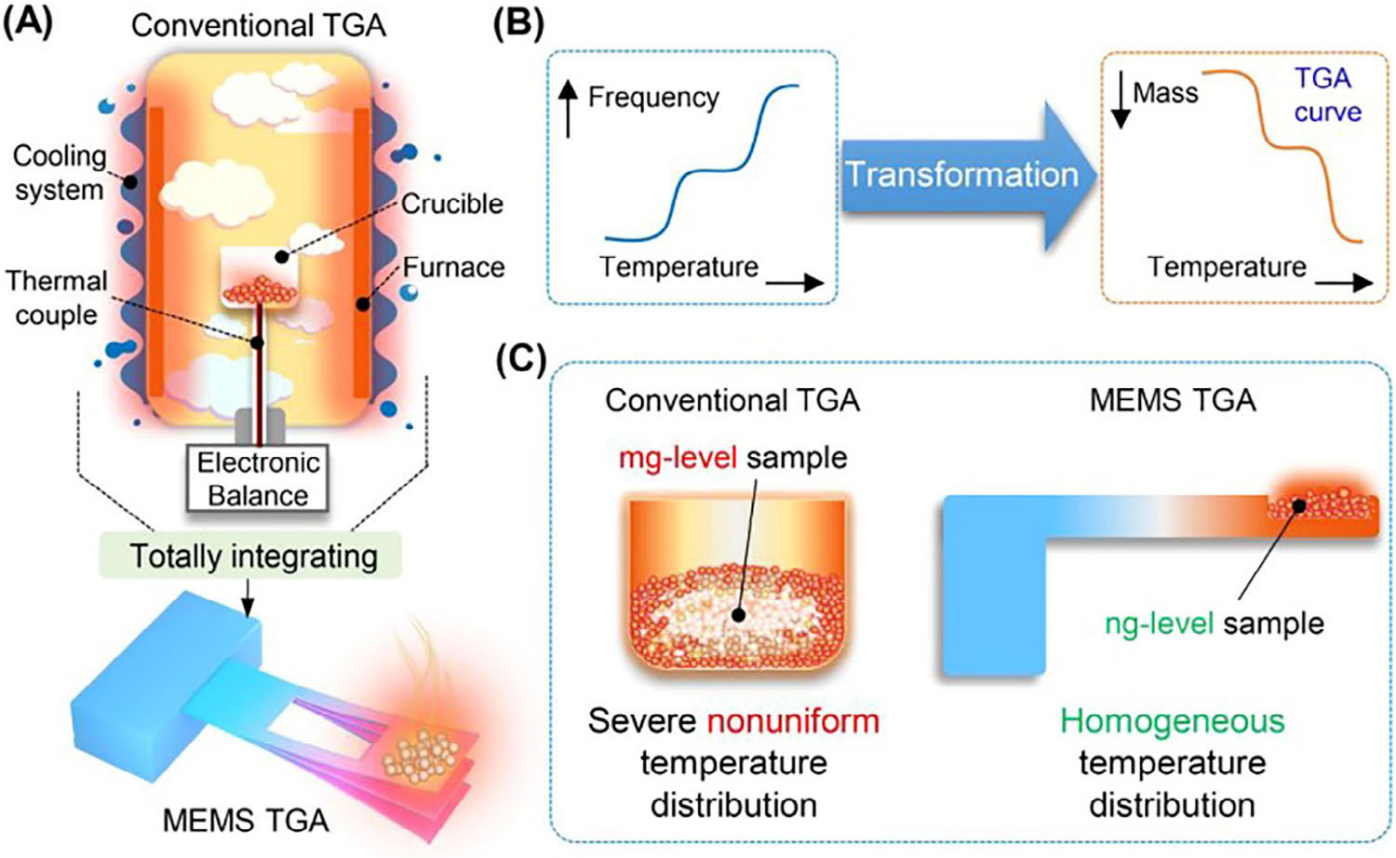
Schematic of MEMS‐TGA technology showing (A) miniaturized TGA components, (B) real‐time mass loss detection and (C) improved heating efficiency. MEMS‐TGA, micro‐electromechanical systems‐based thermogravimetric analysis. *Source*: Figure adapted from Yao et al. [22], Copyright 2021, ACS Analytical Chemistry.

Recent advancements in TGA technology have significantly enhanced its forensic applications, particularly with the development of micro‐electromechanical system‐based TGA (MEMS‐TGA). Unlike conventional TGA, which requires milligram‐scale samples, MEMS‐TGA enables the analysis of nanogram quantities, reducing sample consumption while maintaining analytical precision. Yao et al. [22] reported that MEMS‐TGA, through rapid controlled heating and improved temperature modulation, allows forensic investigators to analyse explosives with unprecedented sensitivity. MEMS‐TGA offers an improved thermal resolution of approximately 0.001°C, compared to the 0.1°C resolution of conventional TGA. Additionally, it enables rapid heating rates up to 10,000°C/min, significantly improving the detection of low‐energy decomposition events in trace explosive residues, a capability crucial for forensic differentiation of similar compounds. Furthermore, MEMS‐TGA enhances laboratory safety by minimizing the risk of accidental detonation, as it requires only minute amounts of energetic material [22]. Figure 4 illustrates the working principle of MEMS‐TGA, highlighting its microcantilever‐based resonator, miniaturized heating system, and real‐time mass loss detection. This schematic demonstrates how MEMS‐TGA achieves superior thermal resolution and forensic sensitivity, making it a groundbreaking advancement in forensic TGA applications.

In addition to MEMS‐based advancements, combined TGA and differential scanning calorimetry (TGA‐DSC) have proven highly effective in forensic investigations. Zeman et al. [21] used TGA to examine the decomposition kinetics of commercial explosives, revealing a linear correlation between the onset temperature of degradation and differential thermogravimetry (DTG) peak positions. This study demonstrated that TGA data could be leveraged to estimate the explosion temperatures of various formulations, aiding forensic experts in reconstructing explosive events. To ensure reproducibility, the sample preparation protocol involved grinding and homogenizing explosive compounds, which minimized variability in decomposition rates. Such findings underscore the role of TGA in predicting the thermal behaviour of explosive compositions under extreme conditions, contributing to forensic reconstruction efforts [21].

Furthermore, thermal analysis techniques such as TGA‐DSC have proven to be quick and safe methods for investigating the thermal properties of primary explosives and other high‐energy materials. TGA sample preparation often involves drying to remove residual moisture and stabilizing the sample under inert gas flow to minimize oxidative interference during thermal analysis. These techniques provide critical data on the thermal characteristics of such materials, thereby contributing to safer handling practices and a deeper understanding of their stability [23].

Another critical aspect of forensic TGA applications is its use in primary explosives and highly energetic materials. Pniewski et al. [23] applied TGA to investigate the thermal properties of selected primary explosives, highlighting the varying stability of different energetic compounds under controlled heating conditions. The results provided forensic scientists with valuable insights into the decomposition pathways and ignition thresholds of these materials, allowing for improved safety guidelines in explosive storage and transport. Additionally, this study emphasized the importance of inert gas environments during thermal analysis to prevent unwanted oxidation, which could lead to erroneous conclusions.

Despite these technological advancements, forensic TGA still faces several challenges, including interferences from environmental contaminants, the complexity of multi‐component explosives and the need for standardized forensic protocols. For instance, explosives mixed with soil, fuels or other environmental debris can alter thermal degradation pathways, leading to overlapping peaks in DTG curves that obscure key forensic signatures. Such interferences necessitate careful sample preparation, such as controlled desorption techniques, to isolate the explosive component from background noise.

The integration of chemometric methods in TGA data analysis has emerged as a promising solution, allowing forensic scientists to extract meaningful information from complex thermal signatures. Compared to DSC, which provides insights into heat flow and enthalpic transitions, TGA uniquely allows for mass loss tracking, making it particularly suitable for differentiating formulations based on thermal decomposition rates. However, unlike differential thermal analysis (DTA), TGA does not provide direct exothermic or endothermic transition data, meaning that complementary DSC or DTA techniques are often required for a complete forensic profile of an explosive material. For instance, forensic identification of binary explosive mixtures (e.g., TATP‐based compositions) often requires both TGA and DSC/DTA to distinguish stabilizer loss from explosive decomposition [24].

To further strengthen the applicability of TGA in forensic science, future research should prioritize enhancing real‐time chemometric integration in TGA‐based forensic analysis to improve spectral interpretation and classification accuracy. Additionally, efforts should be directed towards developing miniaturized, high‐sensitivity portable TGA devices, which would enable on‐site forensic examination of explosives while reducing logistical constraints associated with laboratory‐based methods. Another critical aspect is the standardization of TGA forensic protocols to ensure reproducibility and forensic admissibility in legal proceedings. These advancements will further establish TGA as an indispensable forensic tool, leading to more accurate, reliable and safe analyses of explosive materials.

### 2.3 DSC in Forensic Analysis of Explosives

DSC plays a pivotal role in the forensic analysis of explosives, offering crucial insights into thermal decomposition processes, compatibility assessments and hazard evaluation. DSC operates by measuring heat flow variations during phase transitions, enabling forensic investigators to characterize the thermal stability of explosives, detect exothermic reactions indicative of detonation risks and assess interactions between energetic materials and additives. Unlike traditional thermal techniques, DSC allows for high‐resolution thermokinetic profiling of explosives, identifying unique thermal signatures critical for forensic classification [25, 26, 27, 28].

**FIGURE 5:**
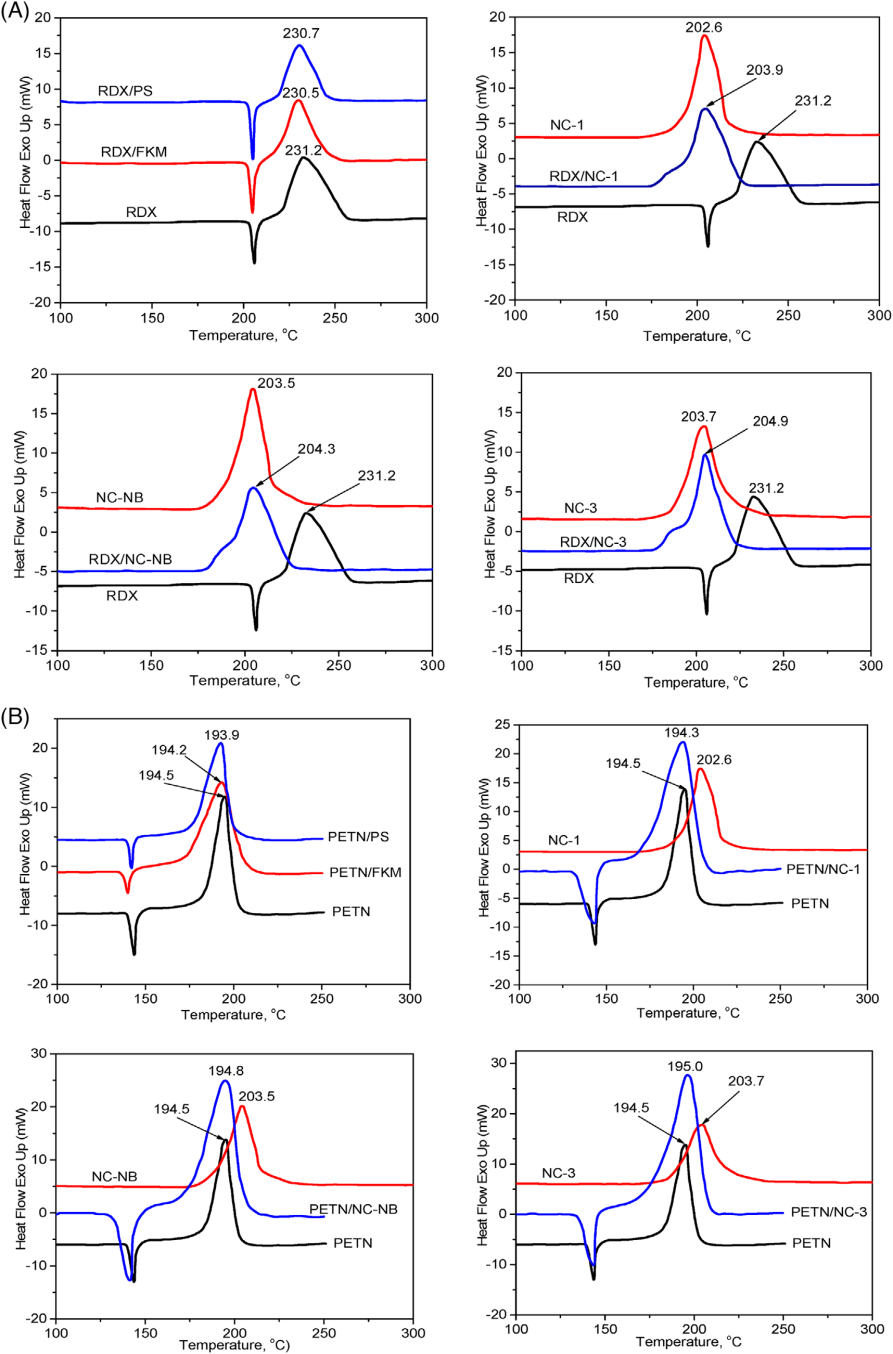
DSC curves of (A) RDX and (B) PETN with various polymeric binders, showing thermal compatibility and decomposition behaviour. DSC, differential scanning calorimetry; PETN, pentaerythritol tetranitrate; RDX, Royal Demolition Explosive. *Source*: Figure adapted from Nguyen et al. [25], published under the CC BY license (MDPI, Polymers).

A notable application of DSC involves evaluating the chemical compatibility and adhesion properties of energetic materials with polymers and binders. This is particularly relevant in the production of polymer‐bonded explosives (PBX), where the thermal interaction between an explosive and its binder influences long‐term stability and detonation characteristics. For instance, DSC has been employed to study Royal Demolition Explosive (RDX) and pentaerythritol tetranitrate (PETN) interactions with polymer matrices, such as polystyrene (PS), nitrocellulose (NC) and fluoroelastomers (FKM). Sample preparation involved finely dispersing explosives within polymer binders followed by controlled thermal cycling. Through DSC analysis, contact angle measurements and vacuum stability tests, it was confirmed that RDX exhibited greater adhesion compatibility than PETN, reinforcing its suitability in PBX formulations [25]. Figure 5 presents DSC thermograms of RDX and PETN mixtures with various polymeric binders, highlighting their thermal compatibility and decomposition behaviours. The observed peak shifts indicate variations in adhesion and thermal stability, essential for assessing binder suitability in PBX formulations.

Beyond compatibility studies, DSC has proven instrumental in investigating the decomposition kinetics of novel high explosives. Hu et al. [26] explored the thermal decomposition of 3,5‐difluoro‐2,4,6‐trinitroanisole (DFTNAN) using DSC and TGA across multiple heating rates. Their study quantified activation energy and decomposition onset temperatures for DFTNAN‐RDX, DFTNAN‐HMX and DFTNAN‐TKX‐50 binary mixtures, identifying critical temperature thresholds at which detonation risks increase. The research provides key safety insights for explosive formulation, ensuring controlled detonation behaviour and compatibility among high‐energy materials [26].

Another critical forensic application of DSC is in assessing the thermal stability of explosives mixed with contaminants. Sun et al. [27] investigated the role of waste engine oil in emulsion explosives, a common scenario in illicitly manufactured explosive devices (IMEDs). DSC analysis revealed that the presence of hydrocarbons lowered activation energy, accelerating decomposition kinetics and increasing explosion hazards under improper storage conditions. This study underscores the importance of DSC in evaluating non‐conventional explosive formulations, where contaminants alter thermal properties in unpredictable ways [27]. DSC has also been utilized in forensic fire and explosion investigations. A study by Wolny et al. [28] demonstrated how DSC and TGA helped reconstruct a fire‐induced explosion scenario involving AN and molten plastics. Their analysis revealed that AN‐polyethylene (PE) and AN‐polypropylene (PP) mixtures exhibited exothermic decomposition above 230°C, leading to the formation of ANFO‐like compositions under fire conditions. These findings provided forensic experts with valuable insight into accidental explosive formation in industrial and agricultural settings, guiding fire investigation protocols [28].

Despite its advantages, DSC‐based forensic analysis presents several challenges. The sensitivity of DSC signals can be influenced by environmental factors, such as humidity, sample contamination and heating rate variations, affecting reproducibility. Additionally, DSC alone cannot provide direct mass loss data, necessitating complementary TGA analysis for a complete forensic profile. Emerging techniques such as high‐resolution DSC (HR‐DSC) and modulated temperature DSC (MT‐DSC) have been developed to enhance thermal sensitivity, allowing for sub‐microgram sample analysis with improved resolution. To further strengthen forensic applications of DSC, future research should prioritize the integration of chemometric algorithms, such as PCA and ML models, to enhance spectral interpretation and improve forensic classification accuracy. Additionally, the development of portable DSC devices is crucial for enabling rapid on‐site forensic examination, bridging the gap between laboratory‐based analysis and field investigations. Standardizing forensic protocols for DSC analysis is equally important to ensure reproducibility and the admissibility of results in forensic investigations. These advancements will solidify DSC as an indispensable forensic tool, allowing for more precise, reliable and legally robust analyses of explosive materials.

### 2.4 GC–MS in Forensic Analysis of Explosives

GC–MS has become a cornerstone technique in forensic science, offering unparalleled sensitivity and selectivity for the detection and characterization of explosive materials. By separating volatile and semi‐volatile compounds through GC and subsequently identifying them using MS, GC–MS enables forensic investigators to obtain detailed molecular fingerprints of explosive residues. This technique is particularly valuable for analysing post‐blast debris, environmental samples and complex forensic matrices, where explosives may exist in trace concentrations or be degraded by environmental factors. Advancements in sample preparation methodologies, such as solid‐phase microextraction (SPME), headspace sampling and solvent extraction techniques, have significantly improved the efficiency of forensic GC–MS applications. Furthermore, derivatization strategies have been increasingly employed to enhance the volatility and stability of thermally labile compounds, ensuring optimal chromatographic separation and detection [29, 30, 31, 32, 33, 34].

Recent studies have demonstrated the adaptability of GC–MS in forensic investigations, emphasizing its role in detecting a broad range of explosive compounds with high specificity. Arce‐Rubí et al. [29] validated GC–MS and GC–NPD methods for the forensic identification of explosive residues, showcasing their ability to enhance the accuracy of post‐blast investigations by detecting a diverse array of organic explosive compounds. The study employed an optimized solvent extraction protocol to recover explosives from various forensic substrates, followed by mass spectral deconvolution to resolve co‐eluting species [29]. Similarly, Valdez and Leif [30] explored the utility of GC–MS in analysing organophosphorus‐based nerve agents (OPNAs), demonstrating how derivatization reactions improve chromatographic resolution and extend the detection range of these hazardous compounds. This study underscored the need for continued advancements in derivatization chemistry to maximize the forensic applicability of GC–MS in nerve agent detection [30].

**FIGURE 6:**
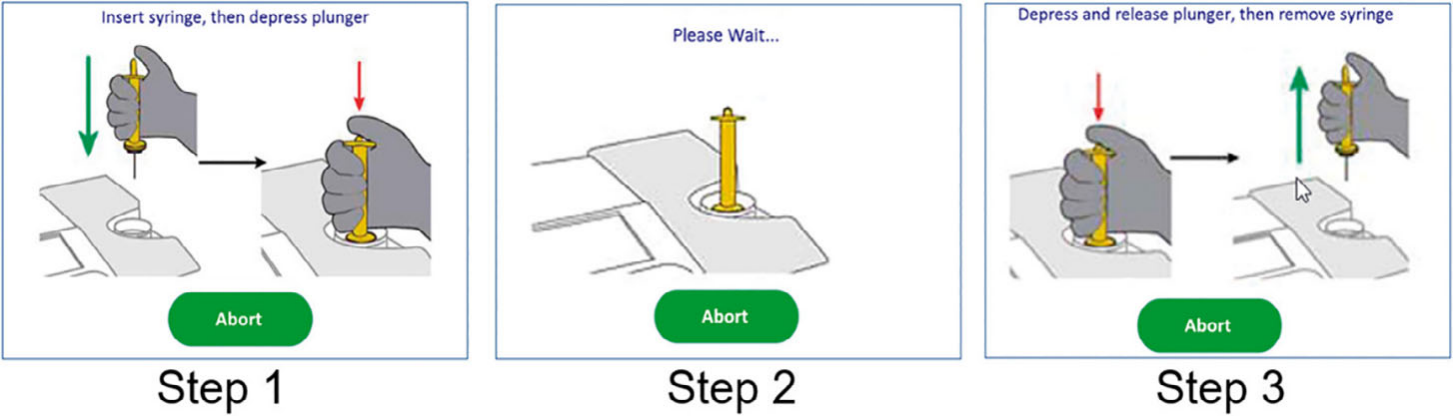
Pictograms for sample introduction in portable GC–MS systems, ensuring accuracy in field analysis. *Source*: Figure adapted from Katilie et al. [31], Copyright 2019, SAGE Publications, Applied Spectroscopy.

Advancements in trace explosive detection have also been explored through specialized GC–MS inlet designs. Katilie et al. [31] developed a method to detect vaporous ammonia, a key marker of AN‐based explosives, by leveraging in‐inlet derivatization to enhance the volatility of ammonium species. This approach improved sensitivity and minimized sample handling, addressing one of the critical challenges in forensic GC–MS applications [31]. Furthermore, the deployment of portable GC–MS instruments has revolutionized forensic fieldwork, particularly in military and counterterrorism operations. Leary et al. [32] discussed the use of field‐deployable GC–MS systems for real‐time identification of toxic chemical agents, highlighting their role in rapid‐response scenarios. The study demonstrated that portable GC–MS instruments, while traditionally less sensitive than laboratory‐based systems, have reached a level of analytical performance that allows for on‐site forensic investigations with minimal sample preparation [32]. Figure 6 presents pictograms used in portable GC–MS systems to guide operators in proper sample introduction during field analysis. These visual aids ensure procedural accuracy, particularly in high‐pressure forensic and military environments where precise sample handling is critical for reliable toxic chemical detection.

**FIGURE 7:**
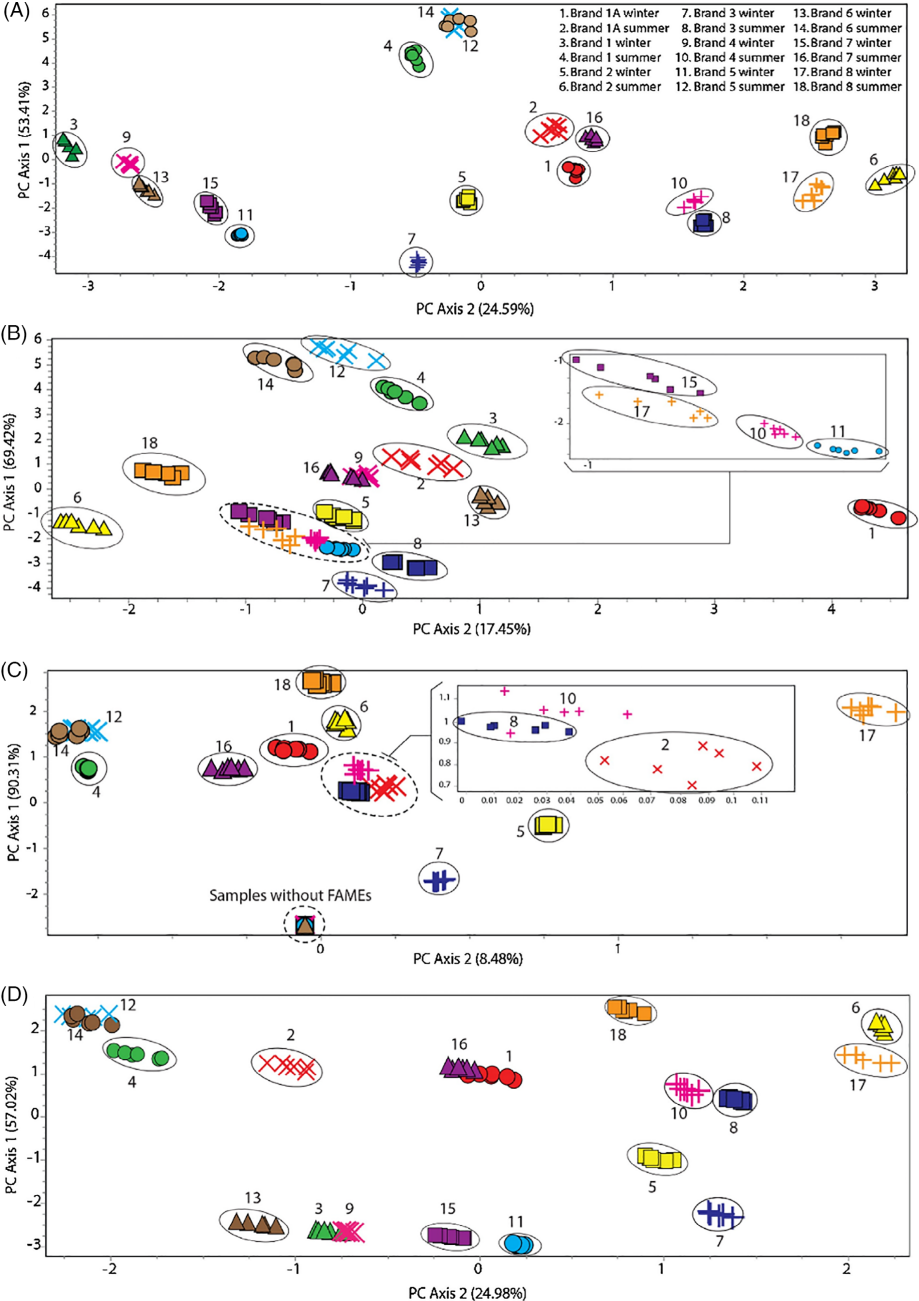
PCA plots showing the classification of ANFO explosive samples based on diesel composition: (A) n‐alkanes, isoprenoids, and fatty acid methyl esters; (B) n‐alkanes and isoprenoids; (C) fatty acids methyl esters; and (D) isoprenoids and fatty acid methyl esters. Each set represents a diffrent selection of diesel compounds used for classification. The correctly classified clusters are shown in circles, highlighting the effectiveness of chemometric analysis in differentiating ANFO formulations. *Source*: Figure adapted from Suppajariyawat et al. [35], Copyright 2019, Elsevier, Forensic Science International.

Beyond environmental and field applications, GC–MS has been employed in waterborne explosives analysis, providing forensic experts with a powerful tool for monitoring contamination and tracking the illicit use of explosive materials. Badjagbo and Sauvé [33] demonstrated the effectiveness of GC–MS in analysing trace explosive residues in water, emphasizing its role in environmental forensics [33]. Similarly, forensic differentiation of explosive formulations has been improved by integrating statistical analysis with GC–MS datasets. Tsai et al. [34] employed multidimensional GC–MS coupled with chemometric techniques to analyse and differentiate plastic explosives based on their unique chemical compositions. This approach allowed forensic analysts to establish chemical signatures for different formulations, enhancing the traceability of explosive sources [34]. In a complementary study, Suppajariyawat et al. [35] used a combined GC–MS and FTIR approach, integrated with chemometric modelling, to classify ANFO explosive samples based on their fuel compositions. Their research highlighted the growing importance of multivariate analysis in forensic science, demonstrating how statistical models can improve the discrimination of explosive materials [35]. Figure 7 presents PCA plots of ANFO supernatant samples, illustrating their classification based on diesel composition. The observed clustering highlights the effectiveness of chemometric approaches in differentiating ANFO formulations, enhancing the forensic traceability of explosive materials.

Despite its strengths, GC–MS faces challenges in forensic applications, particularly regarding the analysis of thermally labile and non‐volatile compounds. Although derivatization strategies have improved the detectability of such compounds, they add complexity to sample preparation and may introduce variability if not carefully controlled. Furthermore, the presence of environmental contaminants in forensic samples can lead to matrix effects, necessitating rigorous cleanup procedures to prevent analytical interferences. Another limitation is the inherent size and operational constraints of portable GC–MS instruments, which, despite recent advancements, still lag behind laboratory‐based systems in terms of resolution and sensitivity. Future developments should focus on miniaturizing high‐resolution mass analysers for portable GC–MS, enhancing spectral deconvolution algorithms to resolve complex forensic matrices and integrating ML models for automated data interpretation. These advancements will further establish GC–MS as an indispensable tool in forensic investigations, allowing for more accurate, rapid and reliable identification of explosive materials in a broad range of forensic scenarios.

### 2.5 X‐Ray Diffraction (XRD) in Forensic Explosive Analysis

XRD is a non‐destructive, high‐precision analytical technique that plays a vital role in forensic explosive analysis by characterizing the crystalline structure, phase composition and purity of explosive materials. By analysing the diffraction patterns produced when x‐rays interact with a crystalline substance, forensic scientists can gain detailed insights into the identification, structural transformations and phase transitions of explosives, which are essential for forensic investigations and safety assessments [36, 37, 38, 39]. Recent advancements in high‐resolution XRD techniques have significantly improved the accuracy of forensic explosive characterization. Schachel et al. [36] demonstrated the integration of XRD into forensic substance analysis, incorporating it into a comprehensive forensic explosive database alongside techniques such as high‐performance liquid chromatography (HPLC), HRMS and x‐ray fluorescence (XRF). Their study analysed pure explosive compounds and synthetic precursors, preparing samples through grinding to uniform particle size to enhance diffraction pattern consistency. This methodological refinement significantly improved the detection of minor crystalline impurities, which is crucial for determining the purity, origin and formulation history of explosive precursors. Such an approach has enhanced the forensic discrimination of illicitly manufactured explosives (IMEs) by providing unique structural fingerprints that differentiate among synthesis pathways [36].

**FIGURE 8:**
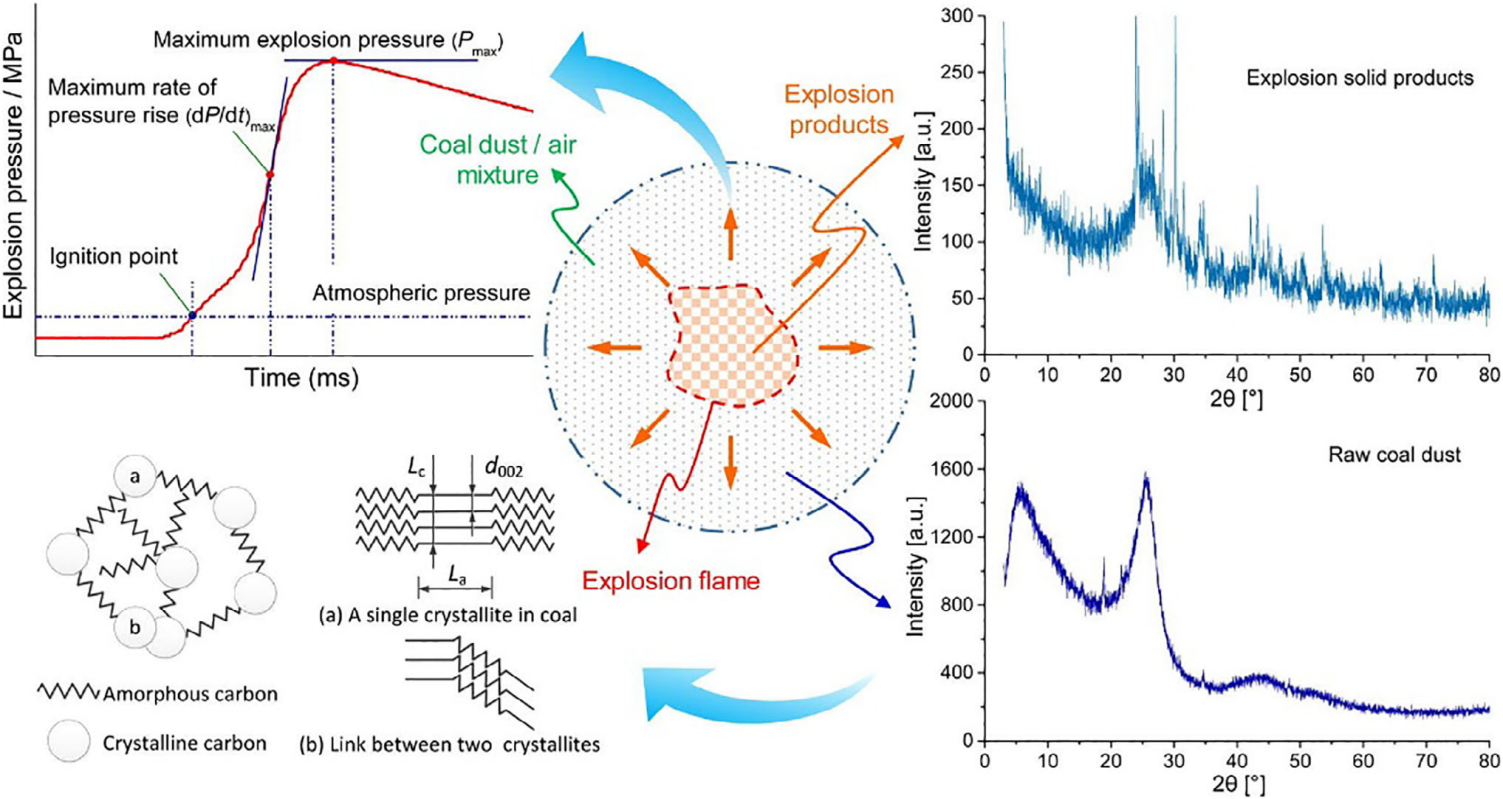
XRD analysis of coal dust explosion residues, highlighting mineralogical transformations. *Source*: Figure adapted from Qian et al. [37], Copyright 2018, Elsevier, International Journal of Hydrogen Energy.

Beyond traditional applications, XRD has been explored for analysing post‐explosion residues to identify structural changes resulting from detonation events. Qian et al. [37] used XRD to analyse coal dust explosion residues, revealing distinct phase transitions in quartz and aluminosilicate compounds. Their research provided insights into the mineralogical transformations occurring in post‐explosion residues, offering valuable forensic indicators for investigating coal mine explosions. The sample preparation involved sieving, drying and moisture removal, ensuring accurate mineral phase detection [37]. Li et al. [39] further investigated the structural evolution of coal dust before and after explosion events, using XRD to examine microstructural changes in carbonaceous materials exposed to detonation conditions. Their findings highlighted how shockwave‐induced modifications in crystallinity can be used to distinguish between pre‐ and post‐blast residues, thereby aiding in forensic reconstructions of industrial explosion incidents [38]. Figure 8 illustrates the forensic application of XRD, highlighting structural phase transitions in post‐explosion coal dust residues. The transformation of carbonaceous materials, evident in the XRD patterns, serves as a key forensic indicator for distinguishing pre‐ and post‐explosion samples, aiding in forensic explosion investigations.

In another notable application, Rajan et al. [38] utilized XRD to characterize a new high‐pressure phase of an energetic material, demonstrating how shockwave‐induced structural transformations influence the behaviour of explosives under extreme conditions. This study correlated changes in XRD patterns and Raman spectra, revealing crystallographic shifts that dictate the energetic response of explosive materials. The processed samples underwent controlled detonation simulations, mimicking real‐world forensic blast investigations to enhance our understanding of phase transitions during explosive events [39].

Despite its advantages, XRD in forensic science faces several limitations. The technique is highly effective for analysing pure, well‐crystallized samples, but its sensitivity decreases when dealing with heterogeneous, amorphous or contaminated residues. Sample preparation techniques, such as chemical washing, separation of amorphous debris and controlled drying, are often necessary to improve signal clarity. Moreover, although XRD provides detailed structural insights, it lacks chemical specificity, often necessitating complementary methods such as GC–MS or chromatography for comprehensive forensic analysis. Integrating XRD with chemometric approaches and multimodal spectroscopy could improve its reliability in forensic investigations by enabling automated phase identification and differentiation of complex explosive mixtures.

Future research should focus on enhancing XRD portability for on‐site forensic applications, improving its resolution for mixed‐phase explosive residues, and developing hybrid analytical workflows that combine XRD with mass spectrometry and chromatography for a holistic forensic analysis. These advancements will further establish XRD as a critical forensic tool for tracing explosive precursors, investigating post‐blast residues and ensuring accurate forensic classifications of explosives. Table 2 below provides a comparative overview of the key analytical techniques discussed in this review, highlighting their respective strengths and limitations.

**TABLE 2:** Comparative strengths and limitations of key analytical techniques for forensic analysis of explosives.

Technique	Strengths	Limitations
IR spectroscopy	Non‐destructive, high sensitivity to organic explosives. ATR‐FTIR enhances surface analysis, and chemometric tools improve classification accuracy. Portable versions enable on‐site forensic identification	Spectral overlaps in complex mixtures reduce specificity. Limited effectiveness in detecting inorganic explosives. Portable devices lack high‐resolution spectral capabilities
TGA & DSC	Crucial for thermal decomposition profiling, explosion risk assessment and forensic characterization of post‐blast residues. MEMS‐TGA enables nanogram‐scale analysis, improving forensic sensitivity	Affected by environmental interferences (humidity, contamination). Limited portability for on‐site forensic applications. Requires careful sample preparation for reproducibility
GC–MS	High sensitivity for detecting trace explosive residues in complex matrices. Portable GC–MS is increasingly used in field‐based forensic applications. Capable of differentiating ANFO and plastic explosives using chemometric models	Requires extensive sample preparation (solvent extraction, derivatization). Portable systems have 30%–50% lower resolution than laboratory models. Non‐volatile explosives require complex modifications
XRD	Provides non‐destructive, high‐resolution crystallographic analysis of explosive precursors and post‐blast residues. Effective in linking precursor materials to synthetic pathways. Useful in forensic coal mine explosion investigations	Less effective for heterogeneous, contaminated samples. Requires additional methods (GC–MS, chromatography) for full chemical specificity. Field applicability is currently limited due to instrument size and setup complexity

Abbreviations: ATR‐FTIR, attenuated total reflectance Fourier‐transform infrared spectroscopy; DSC, differential scanning calorimetry; GC–MS, gas chromatography–mass spectrometry; IR, infrared; MEMS‐TGA, micro‐electromechanical systems‐based thermogravimetric analysis; TGA, thermogravimetric analysis; XRD, x‐ray diffraction.

### 2.6 New Directions for Data Analysis and Chemometrics in Forensic Explosive Analysis

In forensic science, the integration of chemometric techniques has revolutionized data analysis, providing more accurate, reliable and automated approaches for the classification and interpretation of complex chemical datasets. Unlike traditional qualitative assessments, modern chemometric algorithms enable forensic scientists to extract meaningful patterns, identify relationships and classify explosive materials with greater precision. The most commonly used multivariate statistical techniques include PCA, LDA, soft independent modelling by class analogy (SIMCA) and cluster analysis. These methods enhance the ability to distinguish between explosive substances and environmental contaminants, a critical challenge in forensic investigations. In recent years, data preprocessing techniques, such as the Savitzky–Golay smoothing filter, standard normal variate (SNV) transformation and wavelet transformation, have been widely employed to improve spectral quality. These methods correct baseline shifts, reduce noise and normalize spectral data, thereby enhancing subsequent chemometric analyses. Advanced chemometric strategies have also integrated ML models, such as support vector machines (SVMs) and artificial neural networks (ANNs), to develop highly predictive forensic classification models. Such methodologies have been particularly effective in identifying trace amounts of explosives in post‐blast residues and complex environmental matrices, improving forensic detection limits beyond conventional threshold values [16, 17, 40–45]. One widely recognized software tool for chemometric analysis is The Unscrambler, which has been extensively used in forensic chemistry for multivariate statistical analysis of spectral data. The software facilitates exploratory data analysis, clustering and predictive modelling, which are instrumental in forensic decision‐making. In addition to The Unscrambler, other platforms, such as MATLAB, Python‐based Scikit‐learn and PLS_Toolbox, are increasingly being adopted for forensic chemometric analysis, particularly in developing automated detection algorithms for explosives [2, 16, 17, 46–62].

### 2.7 Chemometrics in Explosive Analysis Using IR Spectroscopy

The application of chemometric methods in IR spectroscopy has significantly enhanced forensic explosive analysis, allowing for more precise identification and classification of explosive materials. Unlike conventional spectral interpretation, which relies on qualitative assessments, chemometric algorithms facilitate robust statistical modelling, improving both sensitivity and specificity in detecting explosive residues. Spectral data obtained from IR spectroscopy often consist of high‐dimensional absorbance or reflectance values, requiring sophisticated data processing for meaningful forensic insights. These datasets can be derived from spectroscopic imaging (pixel‐based) or vibrational energy peak tables, necessitating advanced chemometric approaches to filter noise, correct baseline shifts and extract significant chemical patterns for forensic decision‐making [16, 17, 35, 57, 61]. A breakthrough study by Risoluti et al. [57] demonstrated the potential of portable NIR spectroscopy combined with chemometric modelling for the on‐site detection of explosive residues on human skin. Their study employed a MicroNIR spectrometer to analyse spectral data collected from 25 volunteers, optimizing a predictive model capable of distinguishing explosive residues from environmental contaminants. Preprocessing techniques, such as baseline correction, normalization and noise reduction, were employed to minimize individual skin variations, significantly improving classification accuracy. The study reported a classification accuracy of over 90%, highlighting the reliability of chemometric techniques in forensic explosive detection. However, a small sample size and potential environmental variability limit the generalizability of this model, necessitating further validation with diverse population groups and environmental conditions [57].

To enhance the reliability of IR‐based forensic detection, dimensionality reduction techniques such as PCA have been extensively utilized. PCA simplifies spectral datasets by extracting key variance components, thereby improving interpretability while reducing redundancy in high‐dimensional data. Additionally, classification models such as Partial Least Squares Regression (PLSR) and Partial Least Squares Discriminant Analysis (PLS‐DA) have been employed to correlate spectral features with the presence of explosives, mitigating challenges associated with spectral collinearity and noise interference. The use of PLS‐DA enables forensic experts to differentiate explosives from non‐explosive residues based on chemometric modelling, providing a statistically validated forensic framework for real‐time explosive detection [57].

Further advancements in quantum cascade laser (QCL)‐assisted IR spectroscopy have expanded forensic detection capabilities, particularly for trace‐level explosive analysis. Castro‐Suarez et al. [61] explored QCL‐based mid‐IR spectroscopy for detecting explosive residues on diverse substrates, including suitcases and environmental surfaces. Their study successfully identified 2,4,6‐trinitrotoluene (TNT), PETN and RDX, along with volatile precursors such as TATP and 2,4‐dinitrotoluene (DNT). By applying PCA and PLS‐DA algorithms, the researchers effectively distinguished spectral features of explosives from background interference, significantly improving forensic classification accuracy. The ability to detect explosives at ultra‐low concentrations in real‐world scenarios demonstrates the power of QCL‐IRs in forensic applications [61]. These advancements highlight the critical role of chemometric methodologies in IR‐based explosives analysis. By integrating preprocessing, feature extraction and predictive modelling, forensic scientists can achieve more accurate, reliable and real‐time determinations of explosive materials, even in highly contaminated or complex forensic samples. However, challenges, such as spectral interferences, variability in environmental conditions and limitations of field‐portable devices, remain areas for future research. Further optimization of ML‐enhanced chemometric models, along with standardized forensic spectral databases, will enhance forensic detection accuracy, reproducibility and real‐world applicability.

### 2.8 Chemometric Methods in ATR‐FTIR Spectroscopy for Explosives Analysis

ATR‐FTIR spectroscopy, when combined with chemometric techniques, has become a powerful tool for forensic analysis, particularly in the characterization of explosive materials. Recent advancements in ATR‐FTIR‐based chemometric analysis have enabled the precise classification of explosive residues by exploiting their unique spectral fingerprints. By integrating data‐driven models, forensic experts can now distinguish among different sources of explosive materials with improved accuracy. D'Uva et al. [65] utilized ATR‐FTIR spectroscopy alongside chemometric methods to identify the source of AN in HMEs. This study applied PCA and HCA to spectral datasets containing absorbance values recorded at multiple wavenumbers. PCA effectively reduced the dataset's dimensionality by isolating spectral components contributing the most variance, allowing forensic analysts to differentiate between samples with distinct chemical signatures. HCA was then applied to cluster these samples based on spectral similarities, achieving a classification accuracy exceeding 90%. These findings underscore the potential of ATR‐FTIR chemometric models for forensic material traceability, particularly in distinguishing illicitly sourced AN from commercially available counterparts [16].

Beyond AN classification, Banas et al. [17] explored multivariate analytical techniques for post‐blast residue analysis using ATR‐FTIR spectroscopy. Their study incorporated PLS‐DA, a supervised classification technique widely used in spectral data interpretation. The researchers applied preprocessing steps, including baseline correction, noise reduction and spectral normalization, to enhance signal clarity and reduce variability. Feature extraction methods identified key spectral regions corresponding to specific functional groups in explosive compounds. The optimized PLS‐DA model demonstrated high sensitivity and specificity, effectively distinguishing among different classes of explosive residues in post‐blast samples. This work highlights the efficacy of chemometric‐assisted ATR‐FTIR in forensic investigations, providing a robust and reproducible methodology for the rapid identification and classification of explosives [17].

Despite these advancements, challenges remain in ATR‐FTIR forensic analysis, particularly when analysing complex post‐blast residues that contain environmental contaminants or overlapping spectral bands. The integration of more sophisticated chemometric algorithms, such as ML‐based classification models (e.g., SVMs and Random Forest classifiers), could further enhance spectral differentiation and improve forensic classification accuracy. Moreover, recent efforts have been made to develop portable ATR‐FTIR spectrometers equipped with real‐time chemometric processing, allowing for rapid on‐site forensic analysis without the need for extensive sample preparation.

Future research should focus on refining chemometric preprocessing strategies to improve spectral clarity in forensic samples affected by soot, hydrocarbons, or metallic residues. Additionally, standardized chemometric modelling protocols across forensic laboratories would enhance reproducibility and legal admissibility, ensuring consistency in explosive material classification. With continued advancements, ATR‐FTIR spectroscopy integrated with chemometric algorithms will remain a cornerstone in forensic explosive analysis, facilitating faster and more reliable investigative outcomes.

### 2.9 Chemometrics in Explosive Analysis Using Thermal Analysis Techniques

The integration of chemometric methods with thermal analysis techniques, such as TGA and DSC, presents a significant advancement in forensic investigations of explosive materials. These techniques provide detailed insights into the thermal decomposition behaviour, stability and kinetic parameters of explosives, which are crucial for forensic classification and safety assessments. The application of multivariate statistical methods enhances the interpretability of thermal data, allowing for more accurate discrimination of explosive materials based on decomposition pathways, weight loss profiles and heat flow characteristics. Although the direct forensic application of chemometrics in the thermal analysis of explosives is still emerging, promising methodologies can be adapted from related fields. Chauhan et al. [63] demonstrated the integration of TGA with chemometric analysis in soil sample examination, an approach that could be extended to forensic explosive residue analysis. Their study employed PCA and PLS‐DA to extract meaningful thermal degradation patterns from weight loss curves and derivative thermogravimetry (DTG) profiles. By applying pattern recognition techniques, this method facilitated the differentiation of soil samples based on chemical composition and geographic origin [63]. Similarly, in explosive forensic analysis, PCA and PLS‐DA can be used to classify post‐blast residues based on their unique thermal degradation pathways, offering an innovative method for linking explosive materials to their sources.

Further advancements in chemometric‐based thermal analysis are highlighted by Mandal et al. [64], who assessed the spontaneous combustion potential of coal samples using TGA and DSC. Their study incorporated model‐free and model‐based approaches to examine the kinetic parameters of thermal decomposition. Baseline correction and smoothing techniques were applied to improve data accuracy before chemometric modelling. The isoconversional analysis method, a model‐free technique, proved particularly effective for determining activation energy variations at different decomposition stages, eliminating the need for predefined reaction models [64]. This adaptability makes it a promising tool for forensic applications, as it allows for the characterization of unknown or complex explosive compositions.

Additionally, model‐based kinetic analysis segmented the thermal data into multiple reaction steps, facilitating the precise extraction of kinetic parameters, such as activation energy, reaction order and frequency factor. These parameters provide deeper insights into the thermal stability and safety profiles of explosive residues, aiding forensic investigators in reconstructing detonation scenarios and predicting hazardous conditions [64]. The fusion of chemometrics and thermal analysis has expanded the forensic capability of TGA and DSC, enabling a more refined classification of explosive residues. Advanced computational techniques, such as ML algorithms and ANNs, are emerging as powerful tools for enhancing the accuracy of thermal‐based chemometric models. Future research should focus on refining portable thermal analysis systems integrated with real‐time chemometric processing, thereby improving field‐based forensic capabilities. The standardization of chemometric methodologies for forensic applications will further strengthen the reliability and admissibility of thermal analysis in legal investigations.

### 2.10 Chemometrics in Explosive Analysis Using GC–MS

The integration of chemometric techniques with GC–MS has significantly enhanced the forensic analysis of explosives, particularly in the detection, classification and differentiation of complex explosive formulations. GC–MS provides detailed chromatographic and spectral data, which chemometric approaches utilize to detect patterns, classify unknown samples and improve identification accuracy. Given the complexity of explosive residues, multivariate data analysis methods, such as PCA, *k*‐nearest neighbours (*k*‐NN) and LDA, have been employed to extract meaningful chemical signatures and enhance forensic interpretations [34, 35].

One notable study by Tsai et al. [34] demonstrated the effectiveness of chemometric tools in analysing plastic explosives (PEs) through comprehensive GC–MS profiling. Their research focused on chromatographic peak alignment, which was used to correct retention time shifts and improve consistency across samples. PCA was then applied to the aligned chromatographic and mass spectral data to identify key patterns, highlighting variations among different production lots of PE. Classification was further refined using the *k*‐NN algorithm, which assigned unknown samples to their respective production lots based on Euclidean distance metrics. The study achieved a 0% classification error rate, demonstrating the efficacy of chemometric models in distinguishing between closely related explosives with similar chemical compositions. However, the requirement for extensive sample preparation and reliance on controlled laboratory conditions pose challenges for direct application in field scenarios, emphasizing the need for robust, portable GC–MS systems integrated with automated data preprocessing [34].

A significant advancement in this domain was reported by Suppajariyawat et al. [35], who employed a combination of GC–MS, FTIR and chemometric methods to classify ANFO samples based on their fuel composition. Their study processed high‐dimensional chromatographic and spectral datasets, integrating PCA and LDA for comprehensive forensic differentiation. PCA played a pivotal role in identifying clustering patterns corresponding to variations in diesel fuel composition, enabling effective discrimination between different ANFO formulations. This approach facilitated the visualization of intrinsic sample differences and streamlined forensic classification, reducing analytical complexity [35]. Following PCA, LDA was applied to maximize inter‐group separation, correlating the identified clusters to specific diesel brands and seasonal variations. The model successfully classified ANFO samples with high accuracy, emphasizing the utility of chemometric‐driven GC–MS analysis in forensic investigations. Notably, preprocessing steps such as normalization and variable transformation significantly enhanced data quality, reducing experimental variability. The findings of this study have substantial implications for forensic science, particularly in tracing the origins of explosives and linking materials to manufacturers, thereby aiding criminal investigations [35].

Despite these advancements, challenges persist in the application of chemometric techniques in GC–MS‐based explosive analysis. Portable GC–MS instruments, while improving, still struggle to match the sensitivity and resolution of their laboratory counterparts. Additionally, complex sample matrices and background interferences can impact classification accuracy, necessitating advanced preprocessing techniques to improve robustness. Future research should focus on refining automated chemometric algorithms that can operate in real‐time field conditions, enhancing forensic capabilities for rapid and precise explosive identification. Integrating ML approaches with GC–MS chemometric analysis may further improve classification models, offering a pathway towards more adaptive, high‐throughput forensic investigations of explosives.

### 2.11 Chemometrics in Explosive Analysis Using X‐Ray Techniques: XRD

The integration of chemometric techniques with XRD has significantly enhanced the forensic characterization and discrimination of explosive materials, particularly AN, which is widely used in IEDs. XRD provides detailed structural information by analysing diffraction patterns unique to each crystalline compound, allowing forensic scientists to differentiate between pure and adulterated forms of explosives. A study conducted by D'Uva [65] demonstrated the efficacy of XRD in distinguishing among various sources of AN used in explosives. Their research utilized XRD to generate diffraction patterns that served as fingerprint signatures for different AN formulations, revealing variations in phase composition and structural properties influenced by precursor materials and manufacturing processes [65].

To enhance forensic discrimination, chemometric techniques such as PCA and LDA were applied to the XRD data. PCA was instrumental in reducing data dimensionality while preserving critical variance, allowing for the visualization of inherent groupings within the dataset. This approach facilitated the differentiation among pure, commercial and homemade AN samples based on their distinct crystallographic patterns. Following PCA, LDA was employed to classify these samples into predefined categories, optimizing group separation and improving classification accuracy [65].

Preprocessing of XRD data played a crucial role in improving analytical precision. Baseline correction and smoothing techniques were applied to minimize background noise and enhance the clarity of diffraction peaks. Normalization methods were also used to standardize intensity values across different samples, ensuring reliable comparisons. The combination of XRD and chemometric modelling provided a robust methodology for forensic material assignment, yielding highly accurate classification results. Beyond AN analysis, XRD has broader forensic applications, including the identification of crystalline explosive residues in post‐blast investigations and the detection of adulterants in explosive formulations. By leveraging ML algorithms alongside chemometric techniques, forensic experts can automate pattern recognition and enhance the sensitivity of explosive classification models. However, challenges remain, particularly in analysing contaminated samples or mixed‐phase materials where overlapping diffraction peaks may obscure forensic signatures. Future advancements should focus on refining preprocessing strategies, improving reference databases for forensic XRD pattern matching, and integrating XRD with complementary spectroscopic techniques such as Raman and FTIR for a more comprehensive forensic analysis.

## 3 Discussion and Critical Analysis

### 3.1 Effectiveness of Analytical Techniques

This review has critically examined the strengths and limitations of various analytical techniques, including IR spectroscopy, GC–MS, TGA/DSC and XRD, in the forensic analysis of HMEs. Each method contributes uniquely to forensic investigations; however, their practical application, particularly in field conditions, presents notable challenges. IR spectroscopy remains a valuable technique for rapid, non‐destructive molecular fingerprinting of organic‐based explosives, particularly in post‐blast scenarios. However, spectral complexity due to sample contamination and overlapping bands can obscure identification accuracy. Although preprocessing techniques such as baseline correction and normalization mitigate some of these challenges, the sensitivity of field‐portable IR systems remains limited, requiring further advancements in device robustness and chemometric integration. GC–MS is widely recognized for its superior sensitivity and specificity in detecting volatile and semi‐volatile explosive compounds. Despite its effectiveness in controlled environments, its applicability in field conditions is restricted due to the need for complex sample preparation and derivatization for thermally unstable compounds. Although portable GC–MS systems are emerging, they have yet to match the precision and chromatographic resolution of laboratory‐based instruments. The integration of automated sample preparation, enhanced chemometric data processing and ML classification algorithms could improve its usability in forensic investigations.

TGA and DSC provide crucial insights into thermal stability, decomposition behaviour and kinetic profiles of explosives. However, forensic applications of these techniques remain underexplored, with limited literature on their use for HMEs. The challenges of environmental interferences, reproducibility concerns and sample contamination further hinder their widespread forensic adoption. Recent advances in portable TGA/DSC devices and chemometric‐assisted thermal data analysis offer promising avenues for enhancing their forensic relevance. XRD plays a key role in characterizing the crystalline structure of explosive residues, which is valuable for identifying materials such as AN and other precursor chemicals. Although XRD provides detailed structural insights and preserves evidence integrity, its effectiveness is reduced when dealing with contaminated or poorly crystalline samples. To address these challenges, integrating XRD with complementary techniques (e.g., GC–MS, IR) and ML‐driven phase pattern recognition could significantly enhance forensic differentiation and classification accuracy. Overall, these findings underscore the need for targeted research efforts to bridge the gap between laboratory‐based analytical precision and real‐world forensic applicability. By addressing challenges in portability, data complexity and sample preparation, forensic science can enhance the real‐time detection and classification of HMEs, strengthening security measures and criminal investigations.

### 3.2 Impact of Chemometric Methods

Chemometric techniques have significantly advanced forensic analysis by improving the interpretation of complex analytical datasets, particularly in the classification and identification of explosive materials. Among the most commonly applied techniques, PCA, LDA and PLS‐DA have demonstrated significant potential in forensic science. These multivariate statistical methods reduce dimensionality, enhance classification accuracy and extract meaningful patterns from high‐dimensional forensic datasets. However, their application in explosive analysis presents both advantages and challenges that must be critically examined to ensure their reliability in forensic investigations.

PCA is widely utilized for its ability to distil complex datasets, such as spectral and chromatographic profiles, into principal components that retain maximum variance. This technique is particularly beneficial in IR, GC–MS and XRD‐based forensic analysis, where it allows forensic experts to distinguish between explosive materials based on subtle chemical variations. However, one critical limitation is that PCA operates as an unsupervised technique, meaning that although it reveals patterns and clusters, it does not inherently assign classifications to unknown samples. Additionally, reducing dimensionality inevitably results in the loss of some variance, which could affect the forensic accuracy of distinguishing chemically similar explosives. This trade‐off between dimensionality reduction and classification performance must be carefully balanced to maximize forensic applicability.

LDA and PLS‐DA, in contrast, are supervised classification methods that utilize labelled training datasets to optimize separation between predefined groups. LDA is particularly effective when applied to datasets with well‐defined class structures, such as distinguishing different batches of AN‐based explosives. It maximizes the variance among classes while minimizing within‐class variance, leading to highly accurate forensic classification models. However, LDA is sensitive to multicollinearity and data noise, which can distort classification accuracy if spectral or chromatographic data are not properly preprocessed. PLS‐DA, a regression‐based technique, is often preferred for high‐dimensional datasets as it accounts for collinearity and spectral overlapping, making it particularly useful in IR spectroscopy‐based forensic analysis. However, both LDA and PLS‐DA require large, well‐curated training datasets, and their performance can degrade when applied to real‐world forensic samples that exhibit environmental contamination, background noise or varying chemical compositions.

One of the most critical challenges in forensic chemometrics is data preprocessing, which significantly impacts classification accuracy and forensic reproducibility. Preprocessing techniques, such as baseline correction, normalization, Savitzky–Golay smoothing and peak alignment, are essential for improving data quality before applying PCA, LDA or PLS‐DA. For instance, IR spectroscopy data require baseline correction to compensate for instrument drift and environmental interference, whereas GC–MS data require chromatographic peak alignment to correct for retention time shifts across different sample runs. The effectiveness of chemometric classification models depends heavily on these preprocessing steps, and inconsistencies in data preparation can lead to model instability and decreased forensic reliability.

Despite these advancements, the practical implementation of chemometric techniques in real‐time forensic workflows remains limited. One major hurdle is the lack of standardized forensic protocols for chemometric analysis, leading to variability in model performance across different laboratories. Additionally, portable forensic instruments lack the computational power to execute complex chemometric algorithms in real‐time, restricting their field applicability. Recent advances in ML and AI‐driven chemometric models have shown promise in overcoming these challenges. The integration of deep learning with PCA‐based feature extraction and the development of automated chemometric pipelines have the potential to improve classification accuracy while reducing manual preprocessing requirements. However, the forensic validation of these emerging techniques remains an ongoing challenge. To enhance the forensic applicability of chemometric approaches, future research should prioritize (1) optimizing preprocessing pipelines to improve model robustness, (2) integrating AI‐driven chemometric models to enable real‐time forensic analysis and (3) standardizing chemometric methodologies across forensic laboratories to ensure cross‐laboratory reproducibility. Addressing these challenges will enhance the accuracy, reliability and real‐world applicability of chemometric methods in forensic explosive analysis, ultimately strengthening forensic science's ability to detect and classify explosive materials in high‐stakes investigations.

**FIGURE 9:**
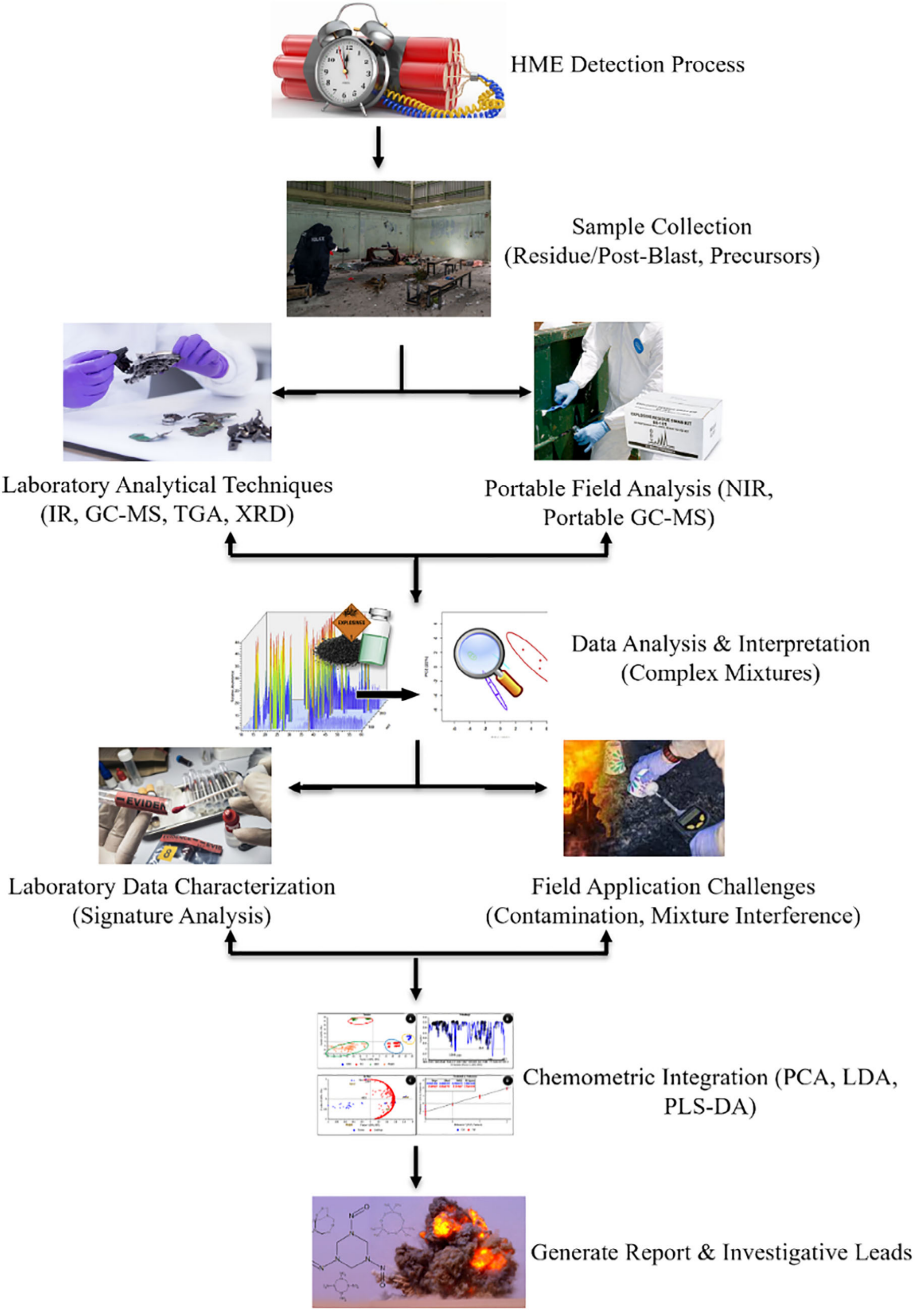
Workflow for the forensic detection and analysis of homemade explosives (HMEs).

Figure 9 presents a detailed flow diagram that outlines the key procedures involved in the forensic analysis of HMEs. The workflow starts with the collection of diverse sample types, including post‐blast residues and precursor materials such as AN and TATP. Field‐specific challenges such as contamination and the complexity of improvised components are addressed through portable analytical methods, including NIR and portable GC–MS systems. Laboratory analysis involves advanced techniques, such as ATR‐FTIR, GC–MS and XRD, to detect and classify explosive signatures accurately. Chemometric tools like PCA, LDA and PLS‐DA play a critical role in managing complex datasets, distinguishing among different formulations and enhancing the reliability of classifications. These analyses culminate in a comprehensive report, providing valuable investigative leads by linking explosive materials to their sources and identifying patterns in precursor usage.

### 3.3 Challenges and Gaps in Current Research

Despite significant advancements in forensic analysis of HMEs, several challenges persist. One of the most pressing limitations is the field usability of advanced analytical techniques such as IR spectroscopy and GC–MS. Although these techniques excel in laboratory environments, portable versions still struggle with sensitivity, resolution and reproducibility when applied to complex HME mixtures in real‐world forensic investigations. The chemical complexity of HMEs also poses a substantial challenge, as they often contain heterogeneous components and environmental contaminants, complicating accurate detection and classification. This issue is particularly problematic in post‐blast scenarios, where explosive residues may be mixed with debris, soil or biological materials, necessitating advanced sample preprocessing and improved chemometric approaches.

Another critical gap in current research is the underutilization of thermal analysis techniques such as TGA and DSC in HME studies. Although these methods have demonstrated value in evaluating the thermal stability and decomposition pathways of conventional explosives, their forensic application to HMEs remains largely unexplored. Future research should focus on adapting TGA and DSC for field‐based explosive residue analysis, particularly by integrating them with chemometric algorithms to enhance classification accuracy. Additionally, although chemometric techniques such as PCA and PLS‐DA have been widely used in controlled laboratory settings, their integration into real‐time portable forensic tools remains limited. To address this, automated, adaptive chemometric algorithms capable of handling environmental variability must be developed to facilitate rapid on‐site forensic investigations. Overcoming these challenges will be crucial in advancing forensic capabilities for the detection, identification and classification of HMEs.

## 4 Conclusion

The increasing threat posed by IEDs, particularly those involving HMEs, underscores the need for advanced, rapid and reliable forensic analysis techniques. This review has examined key analytical approaches, including IR spectroscopy, GC–MS, TGA, DSC and XRD, that provide critical molecular, structural and thermal data essential for explosive identification and classification. The integration of chemometric techniques, such as PCA, LDA and PLS‐DA, has further enhanced the accuracy and interpretability of forensic data, allowing for the effective classification of explosives even in complex, contaminated forensic samples. Despite these advancements, challenges persist in adapting these technologies for real‐time, field‐based forensic applications. The sensitivity and resolution of portable instruments remain limited compared to laboratory‐based counterparts, restricting their effectiveness in on‐site forensic investigations. Additionally, the forensic application of thermal analysis techniques (TGA and DSC) remains underexplored, particularly for the characterization of post‐blast residues and the thermal stability of HMEs. Furthermore, the lack of standardized chemometric protocols across forensic laboratories introduces variability in data interpretation, necessitating the development of unified analytical workflows. Future research should prioritize the advancement of portable forensic tools, the forensic application of thermal analysis techniques and the standardization of chemometric protocols. Strengthening these areas will enhance forensic science's ability to detect, classify and trace explosives with greater accuracy, reinforcing forensic intelligence capabilities in criminal investigations and global security.

## 5 Future Directions

To address the current challenges in forensic explosive analysis, future research should focus on enhancing portable analytical technologies, expanding forensic applications of thermal analysis, standardizing chemometric methodologies and promoting interdisciplinary collaboration. Portable IR and GC–MS systems require improved sensitivity and resolution to match laboratory‐based instruments, enabling real‐time forensic detection in field conditions. The integration of advanced chemometric algorithms, such as ML‐enhanced PCA, LDA and PLS‐DA, could significantly improve classification accuracy and forensic reliability, particularly when dealing with complex explosive residues.

Further research should expand the application of TGA and DSC techniques for HMEs, as the thermal properties of these materials remain underexplored in forensic science. A deeper understanding of thermal degradation pathways will improve post‐blast residue analysis, aiding in tracing explosive compositions and predicting detonation risks. Standardizing chemometric applications is another critical priority, as variations in preprocessing techniques, statistical modelling and spectral analysis across forensic laboratories currently hinder cross‐study reproducibility. Establishing unified analytical protocols will enhance the consistency and accuracy of forensic explosive classification.

Interdisciplinary collaboration among analytical chemists, forensic scientists and statisticians will drive the development of more robust and adaptable forensic methodologies. By fostering research that bridges the gap among analytical chemistry, data science and forensic applications, forensic science can move towards more precise, legally admissible and intelligence‐driven methods for explosive detection and source attribution. Addressing these future research priorities will ensure that forensic science remains at the forefront of security and criminal investigations, ultimately strengthening global safety measures against illicit explosive threats. A summary of key challenges in forensic explosive analysis, along with corresponding affected techniques and future research directions, is presented in Table 3.

**TABLE 3:** Challenges and future research directions in explosive material analysis.

Challenge	Technique affected	Future research direction	Goal
Limited sensitivity in field devices	IR, GC–MS	Develop next‐generation miniaturized spectrometers	Enhance real‐time forensic analysis in the field
Complex mixtures & contamination effects	IR, XRD, TGA/DSC	Improve spectral preprocessing & chemometric correction	Increase accuracy in detecting mixed residues
Underutilization of thermal analysis	TGA, DSC	Expand research on thermal properties of HMEs	Strengthen post‐blast investigation methodologies
Challenges in field chemometric integration	PCA, LDA, PLS‐DA	Embed AI‐driven chemometrics in portable systems	Automate classification & reduce human variability

Abbreviations: AI, artificial intelligence; DSC, differential scanning calorimetry; GC–MS, gas chromatography–mass spectrometry; HMEs, homemade explosives; IR, infrared; LDA, linear discriminant analysis; PCA, principal component analysis; PLS‐DA, Partial Least Squares Discriminant Analysis; TGA, thermogravimetric analysis; XRD, x‐ray diffraction.

## Author Contributions

**Abdulrahman Aljanaahi**: original draft's conceptualization, methodology, data curation, investigation, writing. **Muhammad Kamran Hakeem**: conceptualization, formal analysis, writing review and editing. **Abdulla Aljanaahi**: revision and editing. **Iltaf Shah**: supervision, funding acquisition, project administration, resources, validation, visualization, review and editing.

## Conflicts of Interest

The authors declare no conflicts of interest.

## Data Availability

The data that support the findings of this study are available from the corresponding author upon reasonable request.
